# Community readiness assessment for obesity research: pilot implementation of the Healthier Families programme

**DOI:** 10.1186/s12961-017-0262-0

**Published:** 2018-01-15

**Authors:** Leah A. Teeters, William J. Heerman, David Schlundt, Dawn Harris, Shari L. Barkin

**Affiliations:** 1 School of Education, University of Colorado Boulder, 1134 CO-93 Boulder, CO United States of America; 2 Department of Pediatrics, Vanderbilt University Medical Center, 2146 Belcourt Ave, 2nd Floor, Nashville, TN United States of America; 3 Department of Psychology, Vanderbilt University, PMB 407817, 2301 Vanderbilt Place, Nashville, TN 37240-7817 United States of America; 4 Present Address: 3077 S Jasmine St, Denver, CO 80222 United States of America

**Keywords:** Obesity, Implementation, Dissemination, Community-based, Social infrastructure, Knowledge infrastructure, Environmental infrastructure

## Abstract

**Background:**

This article reports on the development of a systematic approach to assess for community readiness prior to implementation of a behavioural intervention for childhood obesity. Using the Consolidated Framework for Implementation Research (CFIR), we developed research tools to evaluate local community centres’ organisational readiness and their capacity to implement the intervention.

**Methods:**

Four community Parks and Recreation centres from different states expressed interest in piloting an approach for dissemination and implementation of an evidence-based obesity prevention program for families with young children (Healthier Families). We conducted a mixed methods pre-implementation evaluation using the CFIR to evaluate the alignment of organisational priorities with the Healthier Families programme. Written surveys assessed organisational readiness for change amongst organisational leaders, recreation programmers, and staff (N = 25). Key informant interviews were conducted among staff to assess organisational readiness and with community members to assess community readiness (N = 64). Surveys were analysed with univariate statistics. Interviews were transcribed, coded and analysed using inductive and deductive methods of analysis.

**Results:**

Mixed-methods analysis led to the identification of three key domains on which to assess the organisational readiness to adopt a childhood obesity intervention, namely the physical infrastructure, the knowledge infrastructure, and the social infrastructure. The most critical measure of compatibility was the social infrastructure, since obstacles in the knowledge and physical infrastructures could be overcome by the strength of social resources, including the staff’s ingenuity and commitment to a healthier community. This approach guided an assessment of organisational readiness prior to community organisations adopting and preparing to disseminate an obesity prevention community-based program in a wide-range of social and environmental contexts.

**Conclusions:**

Using a comprehensive pre-implementation assessment of the knowledge, physical and social infrastructures in a community is an essential step in effective dissemination for community-based behavioural interventions. Our research found that, when evaluating readiness and alignment, a responsive social infrastructure could provide the capacity to overcome potential barriers to implementation in either the knowledge or physical infrastructures.

**Electronic supplementary material:**

The online version of this article (doi:10.1186/s12961-017-0262-0) contains supplementary material, which is available to authorized users.

## Background

Dissemination and implementation of health-behaviour change interventions requires the translation of effective content to facilitate its adoption community settings. The inherent challenge of aligning (i.e. adapting and contextualising) interventions with the unique environmental characteristics of the target setting necessitates careful assessment of the feasibility, fidelity, quality and sustainability of implementation [1]. Yet, the literature on implementation science for community-based interventions does not yet provide a tested framework for how to successfully adapt and adopt interventions.

The Consolidated Framework for Implementation Research (CFIR) provides a compelling theoretical basis for assessing organisational readiness and capacity to adopt health-behaviour change interventions, though it has been employed less frequently in community-based dissemination research [2, 3]. The CFIR was validated in 2009 and identifies 39 constructs in five domains to be considered when assessing an intervention in a new context [4]. These domains include intervention characteristics, outer setting, inner setting, characteristics of individuals and process. The CFIR has been applied successfully in diverse contexts, most commonly in healthcare settings (e.g. healthcare delivery and process re-design, quality improvement, health promotion and disease management) and health outcomes (e.g. mental health, obesity and blood pressure). The CFIR has also been used to drive data collection and analysis post implementation, with the aim of understanding practitioners’ experiences of the implementation of interventions [5]. The study described in this article reports results from a feasibility assessment aimed at determining how the CFIR can be employed to evaluate alignment and organisational readiness for implementation before introducing an intervention in community settings.

The field of childhood obesity research provides an ideal test case for the application of dissemination and implementation research theory, utilising the CFIR in community settings. In the United States, 17% of children are obese and 33% are overweight. This is a public health crisis that will have lasting consequences, including increased rates of heart disease, diabetes and cancer [6–8]. Obesity is caused by complex factors including individual as well as environmental components. The behavioural factors of obesity could be addressed via community health interventions. However, ensuring successful implementation, adoption and sustainability requires that community organisations have the infrastructure to support community-based behaviour change. We therefore argue that, to make substantive changes to the nationwide epidemic of childhood obesity, the scientific community needs to further develop systematic approaches to the dissemination and implementation of behavioural obesity trials that can be broadly applied without compromising the integrity of the original trial [9]. Trials have shown promising results when interventions aiming to prevent and treat childhood obesity are implemented in community-based settings [10, 11]. This article seeks to expand upon this body of knowledge by focusing on strategies and resources to determine community and organisational compatibility prior to intervention implementation.

Dissemination and implementation research begins with an efficacious intervention. The Healthier Families programme derives from *Salud con la Familia*, a multi-level family-based behavioural intervention designed to be implemented in a community setting, that demonstrated reduction in paediatric obesity in a low-income minority population [12]. *Salud con la Familia* focused on the parent-preschool child pair, recognising both the importance of parents as agents of change for their children as well as the effect of child behaviours on parent behaviours. The intervention consisted of 12 weekly, group-based sessions that taught principles of behaviour change (goal setting, self-monitoring and problem solving) around key content areas important for healthy childhood growth (diet, physical activity, sleep, media use and engaged parenting). The intervention was delivered in local community centres, leveraging the built environment for health. To translate the efficacy of *Salud con la Familia* from one local context to a generalisable and sustainable approach to reducing childhood obesity, the next step was to develop an effective strategy for dissemination and implementation in other communities. The first stage of dissemination and implementation is assessing feasibility in diverse communities. This intervention relies upon the infrastructure of the local setting, utilising community centres and their staff to promote healthy lifestyles. Due to this reliance and partnership with the community centres, the pre-implementation stage assesses the compatibility of the community centre, as well as the population it serves.

The purpose of this study was to adapt *Salud con la Familia* so that it could then be adopted by a wide range of communities. This article reports on the development of a systematic approach for assessing organisational/community readiness and alignment prior to the implementation of the newly adapted Healthier Families programme – a family-based behaviour change programme to reduce paediatric obesity. Using the CFIR, we developed a feasibility assessment with suggestions to guide future dissemination and implementation efforts for health behaviour change interventions in the community.

## Methods

The process of determining alignment for dissemination of the Healthier Families programme utilised a mixed-methods approach to assess organisational and community readiness using the CFIR as a theoretical guide. To assess organisational readiness to adopt the Healthier Families programme, surveys were conducted using quantitative measures of organisational readiness and semi-structured key informant interviews with several members of each Parks and Recreation facility. Community readiness and interest in this type of community programming was assessed through semi-structured interviews with community members identified by our partner sites. The surveys captured quantitative data and the interviews provided qualitative details. Survey and interview instruments were aligned to the CFIR domains of intervention characteristics, outer setting, inner setting, characteristics of individuals and process. All participants who completed the survey or participated in a semi-structured interview signed an informed consent document prior to participation. The Vanderbilt University Medical Center Institutional Review Board approved this study.

### Identification of partner sites

Our research team recruited potential community partners via the National Recreation and Parks Association listserv, which we used to share a description of the Healthier Families programme. After Parks and Recreation programmes expressed interest, phone calls with the self-identified Parks and Recreation programme leaders were then conducted to determine potential for compatibility and availability to commit to participating in the 2-year project with monthly meetings. The convenience sample consisted of sites that met an initial inclusion criteria that included (1) an active lead community partner being identified; (2) serving parents and young children; (3) prioritising community health; and (4) supported by their recreational leadership to participate in a 2-year community-academic pilot. From this process, four sites emerged in the states of Georgia, Michigan, Florida and, initially, Minnesota. During the course of the first year, the physical infrastructure changed in Minnesota, making the collaboration no longer viable. However, Minnesota staff suggested the Parks and Recreation centre in Nevada as a potential replacement. Our team repeated the evaluation process with Nevada to confirm both interest and availability.

### Organisational readiness

The leadership teams at each of the Parks and Recreation departments identified key staff in their organisations to participate in the organisational readiness assessment. Potentially eligible staff were then contacted directly by the study team to gauge their interest in participating, focusing specifically on the confidentiality of their responses. Of 32 parks and recreation staff invited to complete the modified organisational readiness to change assessment, 32 consented to participate and 25 completed the survey (Table 2). Parks and Recreation staff participating in the survey represented leadership staff, programmatic staff and administrative staff.

The quantitative assessment modified an existing instrument developed by Helfrich et al. [13]. This modified survey (items available upon request) consisted of 25 items and assessed respondent agreement regarding organisational readiness to adopt the intervention in the domains of Mission Alignment (1 item), Leadership Culture (6 items), Leadership Style (6 items), Readiness for Change (4 items), Role Clarity (4 items), and Resources (4 items). Response options for each item were on a 5-point Likert scale from 1 (strongly disagree) to 5 (strongly agree). Results are reported as the average score (possible range 1–5) for each sub-domain. All respondents had complete data on the survey items.

Key staff members were subsequently invited to participate in face-to-face key informant interviews conducted by the study team during a site visit. The semi-structured interviews presented an overview of the 12 core modules (materials sent prior to the interview), solicited input on the process of delivering these modules within the practical context of their space and time allocations, and asked for feedback on both the process and content with consideration to the individual family, the recreation centre leader, and the recreation centre as a whole. Interviews took between 30 and 45 minutes to administer; they were audio-recorded and then transcribed. See Additional file 1 for full interview questions.

### Community readiness

For all four sites, staff identified 10 families with young children to participate in key informant interviews. The primary caregiver then participated in a phone interview with a Vanderbilt research assistant.

The inclusion criteria for families were (1) parents of 3- to 5-year-old children; (2) English-speaking, and (3) who had phone access. Semi-structured interviews with parents provided qualitative data for establishing an understanding of families’ activities, interest in the Healthier Families programme topics and potential barriers to participation. The general outline for the interviews presented an overview of the 12 core modules, solicited feedback on both the process and content, and identified potential barriers to participation. See Additional file 1 for full interview questions.

### Data analysis

Descriptive statistics (mean and standard deviation, percent or median, and inter-quartile range (IQR)) were used to summarise responses to the survey items. While each site received anonymous and individualised reports regarding the responses to survey items at their centre, we present them in aggregate here to preserve confidentiality. The survey was administered electronically and data were stored in a secure REDCap database. The results of the survey were analysed to determine if there was a baseline of organisational compatibility before moving forward.

The interviews were audio recorded and the recordings were transcribed by a professional transcription service. Analysis included both inductive and deductive coding systems. A deductive coding system was developed based upon the CFIR domains, as it applied to the family-based community centred context of the Healthier Families programme. This coding system was then applied to describe, sort and analyse the interviewee quotes. The coding system captured discussion related to key questions about adaptation to specific communities. For the staff interviews, there were six major groups of codes, with most groups having several subcategories (Additional file 2). Each quote could be given 1–3 different codes.

Inductive methods [14] were used to analyse the data from the surveys and key informant interviews. Emergent themes relating to elements of the infrastructure that were particularly relevant to the CIFR’s application in a community-based setting were identified in this process. These themes were integrated into a dashboard that visually represented each of the key organisational and community readiness domains. Through team consensus, an assessment of each of the domains based on the perceived compatibility with the Healthier Families programme was determined, and a colour-coded designation was assigned to each category (Green: strength of the programme with no anticipated barriers to implementation; Yellow: potential or minor barriers to implementation identified; Orange: significant barriers to implementation identified without potential solutions).

## Results

The four Parks and Recreation sites that participated represented four distinct communities, creating variability in both the organisational characteristics and the community’s sociodemographic characteristics (Table 1). Some of the key organisational differences were reflected in the types of programming that the Parks and Recreation centres offered. For example, the Florida site focused primarily on sports leagues for school-aged children and tutoring programmes, whereas the Michigan site focused primarily on physical activity classes for community members of all ages.

**Table 1:** Description of each Parks and Recreation site. Monthly climate data were obtained from the National Climatic Data Center [21]. Demographic data were obtained from 2010 United States Census Data [22]

Michigan		
Programmes offered • Yoga • Karate • Boot camp • Dance • Acrobatics • Afterschool programme • Art	Climate • Average temperature: 23 °F • Average low temperature: 17 °F • Average high temperature: 30 °F	Demographics Population: 114,297 • White: 61.2% • Black: 23.7% • Hispanic or Latino: 12.5% • Asian: 3.7% • American Indian: 0.8% • Median household income: $36,054
Georgia		
Programmes offered • Youth basketball • Cheerleading • Zumba • Yoga • Line dancing • Senior drama class • Nutrition Class	Climate •Average temperature: 61 °F • Average low temperature: 51 °F • Average high temperature: 70 °F	Demographics Population: 691,893 • Black: 54.3% • White: 33.3% • Hispanic or Latino: 9.8% • Asian: 5.1% • American Indian: 0.84% • Median household income: $50,856
Nevada		
Programmes offered • Dance with baby • Youth boot camp • Yoga & Tai Chi • Art • Music • School prep • Language	Climate • Average temperature: 63 °F • Average low temperature: 48 °F • Average high temperature: 77 °F	Demographics Population: 257,729 • White: 76.9% • Hispanic or Latino: 14.9% • Asian: 7.2% • Black: 5.1% • American Indian: 0.7% • Median household income: $64,489
Florida		
Programmes offered • Afterschool programme • Tutoring programme • Computer lab • Internet access • Teen arts • Fitness & personal Training • Karate	Climate • Average temperature: 77 °F • Average low temperature: 70 °F • Average high temperature: 85 °F	Demographics Population: 107,167 • Black: 76.3% • Hispanic or Latino: 22.0% • White: 18.3% • Asian: 0.6% • American Indian: 0.2% • Median household income: $42,040

### Survey results

The responses from the organisational readiness to change survey indicated that staff thought that they had the capacity, resources and enthusiasm (Table 2). The average (SD) scores for each of the remaining sub-domains were Leadership Culture 4.2 (0.7), Leadership Style 4.2 (0.7), Readiness for Change 4.2 (0.7), Role Clarity 4.3 (0.8), and Resources 4.1 (0.6). The majority (93%) of staff surveyed reported that they believed that ‘current programmes and member experiences can be improved’. Similarly, 22 (88%) staff members reported that the community centres ‘are willing to try new things’. When specifically considering the implementation of the Healthier Families program, 22 (88%) staff members reported their team ‘will share the responsibility for the success of the Healthier Families programme’.

**Table 2:** Responses from the organisational readiness to change assessment. The survey consisted of six domains, scored on a Likert scale from 1 (strongly disagree) to 5 (strongly agree). Mean Likert scores are presented with standard deviation for the overall survey, and then by study site. Individual study site is blinded to prevent identification of specific communities

	Overall	Site 1	Site 2	Site 3	Site 4
	N = 25	N = 8	N = 5	N = 6	N = 6
Mission alignment	4.92 (0.3)	5.0 (0.0)	5.0 (0.0)	4.7 (0.5)	5.0 (0.0)
Leadership culture	4.2 (0.7)	4.5 (0.4)	4.1 (1.0)	3.8 (0.8)	4.5 (0.6)
Leadership style	4.2 (0.7)	4.6 (0.4)	3.8 (1.0)	3.8 (0.8)	4.3 (0.4)
Readiness for change	4.2 (0.7)	4.6 (0.4)	3.9 (0.7)	3.8 (0.8)	4.5 (0.4)
Role clarity	4.3 (0.8)	4.5 (0.5)	4.1 (0.8)	3.8 (1.3)	4.6 (0.5)
Resources	4.1 (0.6)	4.0 (0.7)	4.2 (0.6)	3.8 (0.7)	4.4 (0.4)

There was near uniform agreement that each of the Parks and Recreation centres were ready to adopt the Healthier Families programme. When asked if the Healthier Families programme aligned well with the mission of the local Parks and Recreation department, 23 (92%) participants responded ‘strongly agree’ while 2 (8%) participants responded ‘neither agree or disagree’.

### Interview data

**Fig. 1:**
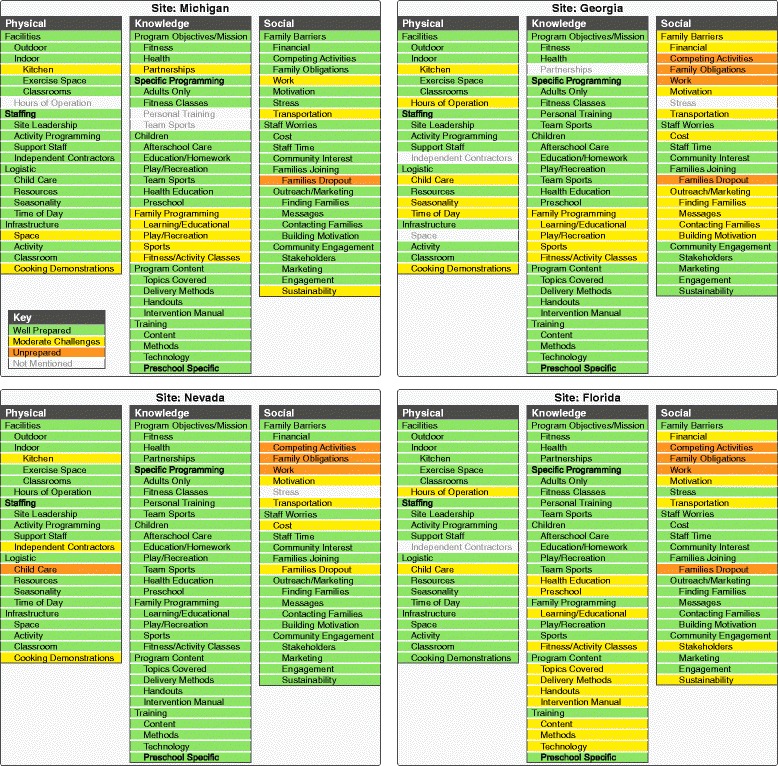
Readiness for implementation. Green indicates that the site is well prepared for implementation, yellow indicates that there may be moderate challenges with specific elements of implementation, and orange indicates that the site was not yet prepared for specific elements of implementation

Using a deductive (i.e. based on CFIR) and inductive (i.e. based on responses from key informant interviews and surveys) approach to analyse both interview data from both the families and the recreation staff, three key domains emerged on which to assess organisational and community readiness to adopt the Healthier Families programme, namely the physical infrastructure, the knowledge infrastructure and the social infrastructure. In each of these categories, there were several sub-domains that were assessed for alignment with the Healthier Families programme (Fig. 1).

Figure 1 helps to identify areas of strength and areas for support, suggesting how the two can work together. For example, in Georgia, in the domain of social infrastructure, ‘family barriers’ and ‘outreach and marketing’ were both identified as potential challenges. Yet, within the same domain, ‘community engagement’ was identified as high and ‘staff worries’ as minimal. This indicates that the community centres could leverage their strong community engagement in the process of outreach and marketing. Similarly, potential ‘family barriers’ could be overcome by the ingenuity of the staff’s ability to accommodate a family’s potential obstacles to participation.

### Physical infrastructure

For the purposes of the Healthier Families programme the compatibility of physical infrastructure included the availability of physical space to accommodate the classes, access to a kitchen for cooking demonstrations, and opportunities for community families with preschool age children to engage in physical activity.

#### Compatibility

The Healthier Families programme aims to serve approximately 10 families at a time. The course involves interactive content, so needs to take place in a space where participants can comfortably move about the room and where facilitators can write on the board. Every Parks and Recreation department that we worked with had suitable classrooms and recreational facilities. Furthermore, since physical activity is a focus of Healthier Families, indoor space, outdoor space and physical fitness programme offerings were an important component of alignment. Parents that were interviewed at all four sites frequently commented that these spaces were commonly used by the community for a wide range of purposes, including things like team sports, ballet and jazz classes, swimming classes, and outdoor playgrounds and park spaces. In Michigan, a parent shared “*my kids have taken a lot of classes through the Parks and Rec facility*”. In Florida, parents shared that, in addition to regularly using recreation facilities for physical activities such as “[going to the] *park, playing sports outside,* [and playing] *basketball*”, they also valued that “*you’re able to go in and use their computer areas,* [and] *they have internet or family time where you’re able to teach your child how to surf the internet safely*”.

#### Challenges

Although there were adequate spaces for instructional sessions and plenty of space and opportunities for participants to engage in physical activity, each city’s recreation centre presented some degree of physical constraints. At every site (which consisted of multiple community centres within one community) not all of the community centres had full kitchens, limiting the capacity to prepare food. This is notable considering that families interviewed cited the cooking classes as a significant programmatic interest. Participants shared that even if the cooking components cost, it would “*totally be worth it*”. Similarly, scheduling was also commonly identified as a conflict, whereas the community centres were not always open during the times that families shared were most convenient for them: “*usually when they're offering activities I'm still at work, and she's obviously still at the afterschool programme*”. When the recreation centre hours aligned, there were often competing needs for classrooms. A site coordinator expressed the challenges of finding physical space: “*We're very over loaded. The centre, like you've probably seen with other centres, it's really small. We have the gym, we have two decent sized rooms. Literally, our nights, after 3:00 it's booked and then weekends it's booked*”.

#### Response to obstacles

Despite these limitations, community centre staff members were quick to generate possible solutions. For example, in Michigan, where only two of four recreation centres had full kitchens, staff suggested the ability to “*make a kitchen space*”, bringing in hot plates and necessary utensils to create a kitchen and working creatively with the schedules to identify space for classes. Similarly, the Georgia and Nevada staff shared that their kitchen space was rather small and could not accommodate a class of 10 participants; however, they were quick to follow-up in saying that it would be easy to do the preparation in an adjacent room and move between spaces. Similarly, the recreation staff who shared the challenges of coordinating physical space, affirmed that, despite the challenges, “*I can still find a space one night a week*”.

### Knowledge infrastructure

In order to implement the Healthier Families programming, facilitators required familiarity with instructional methods and content related to health. Facilitators also required experience with adults and pre-school learners.

#### Compatibility

The staff and parents at all four sites identified an alignment between the goals of the Healthier Families programme with both the vision of Parks and Recreation as well as the values of the local community. The staff at all sites consistently valued lasting relationships with families that were oriented around health and wellness, and valued partnerships such as Healthier Families that could support this aim. For example, a Michigan community centre staff member shared that their team was “*really focused on health, and wellness and trying to provide that for people in the community*”. Another staff member said, “*not just while they're here with us, but also considering how do we get them to take that then beyond, and make that part of their lifestyle*”. Community members at all four sites similarly displayed enthusiasm for the alignment of Healthier Families to the goals and values of the community. One person said that the Healthier Families programme, “*seems like a very good programme to offer in the community. Everyone is very concerned about health and we want to eat right and we want to do right*”, confirming that Healthier Families “*would be welcomed with an open-armed feeling*”.

#### Concerns

At all four sites, one of the main issues raised as a potential barrier to implementation was the relative lack of existing programming for pre-school children, along with a lack of staff training to work with children in that age group. Several of the sites had close ties with preschools, yet the programming offered by the Parks and Recreation department did not include many opportunities for this age group aside from supervised play. Similarly, the community centres did not have significant programming for parents and young kids to engage in activities together. However, staff did indicate an interest in developing programming for this age group: “*I wish we did after care for preschoolers. That would be so much fun. I immediately, when I think about school, I think about kindergarten to high school*”.

#### Response to obstacles

The staff at all sites were excited to obtain the necessary training and support, to work with pre-school aged children as they were “*very interested in learning something new to implement into our community*”. The community centre leadership spoke on behalf of the staff, confirming that the limitations in past experience would not be obstacles: “*I think with the proper training we would be ready… I think they would be ecstatic about it if it's presented and done in the right way*”. The leadership went on to assert that “*I think in order to make that work you really only need one or two good staff*”, affirming that, though there may be some areas for growth in terms of knowledge and experience, the commitment of a few dedicated staff and leadership could overcome identified obstacles and weaknesses with concerted plans for training and organisational learning.

Moreover, staff at the local recreation centres expressed confidence and enthusiasm about the prospect of learning new skills. The staff were excited about training opportunities and open to using technology. They recognised the need for ongoing training and supervision during programme implementation.

### Social infrastructure

The social infrastructure refers to the social components of an organisation, including the leadership, staff and contracted workers, the community members, and the relationships between the organisational staff and the community members, including elements such as outreach and engagement.

#### Compatibility

At all four sites, the commitment, ingenuity and leadership of the Parks and Recreation staff, the relationships between the recreation centres and the local community members, and the enthusiasm and social network of the local community are significant assets. In Michigan, the greatest area of strength, both as a community centre and as a compatible site for Healthier Families, was the commitment and energy of the staff, who “*are really invested*”. Staff members shared that their leaders were organised, had strong initiative, generate a sense of teamwork, and provide vision and follow-through. In Georgia, a community member asserted that they imagined that the Healthier Families implementation “*is going to work great*” due to alignment of mission and the relationships the community centre has with families. The staff at this location affirmed that, “*we have the organisational support*”. Moreover, community members and Parks and Recreation staff expressed a strong sense of community, expressing that there may be other potential partners in government, not-for-profit and business sectors that would make good partners in making the Healthier Families programme a success.

#### Concerns

A concern that the staff members at all sites shared regarding the Healthier Families programme was the length of the time commitment to complete the programme (12 weeks). The recreation centres shared that typically the programmes offered at the community centres are only a few weeks long: “*I sometimes think* [our classes] *are a lot, and they only last five weeks. To double that, and add two more…*” Families similarly reported that the length of the programme was longer than they typically commit to.

In Michigan, community members expressed a desire for the programming to be inclusive of differing family composition, asking that the Healthier Families programme “*take into account how non-traditional families are nowadays*”. Parents suggested “*focus on single parent families*”, as well as inclusion of multiple generations such as grandparents. Moreover, some families shared that they cared for foster kids, who often did not have a stable sense of a nuclear family.

Some community members interviewed valued the content, but did not perceive it as relevant to them specifically because they are already proficient in the content: “*It sounds like a great programme. I just feel like it's providing a skill set that I already have a pretty solid base in*” or “*I think at least for our child because he’s real active anyway*”. Often, these were the families with available time. Those who might have been more ‘in need’ of the content had less availability.

#### Response to obstacles

When discussing the concern of the length of the programme, the staff was quick to generate solutions, sharing ideas for recruitment, community engagement, and motivation, which could mitigate the risk of dropout. Staff also expressed a desire to meet the needs of the community members that they were serving, and although they did not explicitly reference the inclusion of diverse family structures, the staff’s continued expression of intent to “*meet the needs and embrace the values of community members*” indicate that they would be accommodating and inclusive as these issues were brought to their attention.

### Considerations of alignment

At the Florida site, the community’s priorities and needs (i.e. social infrastructure) were focused on educational attainment rather than the priority of family health that would have been essential to implement the Healthier Families programme. Parks and Recreation leadership and staff demonstrated a deep commitment to meeting the needs of their community. Consequently, the majority of the existing programming was focused on educational enrichment (e.g. afterschool programmes, computer labs, tutoring, internet access), rather than health-related activities (e.g. nutrition or physical activity classes). One recreation leader reported that children “*can come straight from school to here and get help with* [their] *homework and that gives the mom more time to not have to rush home from work in the rush hour traffic…. We've made it a little more convenient for those parents that really want those kids to get involved but have those hiccups with the job and the transportation and those types of things. We've offered that service at almost a zero cost in price what we're charging them*”. In our interviews with parents, they talked about the stress of being a working parent and shared that they had long working hours. In the above quote, we see how the Parks and Recreation staff were aware and responsive to the needs of their community.

## Discussion

### Systematic approach to determine goodness of fit for dissemination of the Healthier Families programme

After a thorough evaluation of the quantitative and qualitative data that included both the research team, the local staff at the Parks and Recreation departments, and families with young children who lived in those communities, goodness of fit in the categories of physical, knowledge and social infrastructure could be more easily identified. In three of the sites (Michigan, Georgia, Nevada) there was sufficient alignment to move forward with dissemination. The coding framework guided a qualitative discussion with regards to alignment, with a focus on potential barriers. All sites had potential barriers and challenges; however, the determining factor of alignment was in how sites responded to potential conflicts. For example, in Michigan, Georgia and Nevada, staff presented potential solutions to perceived challenges. In these sites, the staff and community prioritised the mission and vision of Healthier Families. In contrast, while the Florida site had some physical, knowledge and social infrastructure, the focus and priorities of those systems were not fully aligned with the mission of Healthier Families and the identified priority of their community members. This was evident in the response to both survey and interviews, as well as response time to data collection efforts. This systematic approach identified that the Florida site did not have alignment with the programme at the time of the assessment and will be repeated in the future if there is interest.

### Implications

We provide a systematic process and application using the CFIR framework to assess community readiness and alignment for dissemination of an evidence- and community-based behavioural intervention for childhood obesity. Using the theoretical underpinnings from the CFIR, our mixed methods approach inductively identified three key domains that should be evaluated as new communities adopt evidence-based obesity interventions, namely the knowledge, physical and social infrastructure. Our experience with this process suggests that a careful pre-implementation evaluation of potential barriers to implementation in these domains is essential for effective implementation and dissemination, with direct input from both Parks and Recreation leaders and community members. As the Parks and Recreation departments who participated sought to address issues identified by the pre-implementation assessment, it became clear that no site had all requisite elements in place. However, our data suggest that the leading measure of compatibility was the social infrastructure, whereby obstacles in the knowledge and physical infrastructures could be overcome by the strength of social resources, including the staff’s ingenuity and commitment to a healthier community. Our findings suggest that future research focusing on the implementation of community-based behavioural interventions for obesity require an analysis of the compatibility of the social infrastructure.

During the course of this process, it became clear that two of the Parks and Recreation departments that initially expressed interest could not participate, providing meaningful insight into what is needed for readiness of implementation and dissemination. In one case (Minnesota), it was due to a lack of available physical infrastructure; in the other (Florida), it was due to the social infrastructure with a lack of aligned priorities between the recreational leaders and the community and the availability of recreation facilitators with time to be trained for programme implementation. In both cases, it necessitated the deferral of implementing the Healthier Families programme.

This study advances the field of dissemination and implementation research by developing and pilot testing a systematic approach prior to disseminating a community- and evidence-based obesity programme, building on a well-established theoretical framework (i.e. CFIR). The majority of behavioural interventions for paediatric obesity do not adequately report elements necessary to assess external validity, including setting level inclusion criteria and representativeness, characteristics regarding intervention staff, implementation/fidelity to the intervention content, or programme sustainability [15, 16]. By using a dissemination and implementation framework, this study explicitly translates those key measures necessary for assessing external validity into actionable assessments that facilitate the adoption of the intervention in a wide range of social and environmental contexts. Furthermore, our findings are consistent with previously developed conceptual frameworks for applying public health policy in childhood obesity research, recognising both content- and process-related barriers to implementation of efficacious programmes in multiple social contexts [17]. While little evidence has been generated in the dissemination and implementation of childhood obesity research, there are key similarities between our findings and those reported in adult dissemination and implementation trials for obesity research. For example, Kozica et al. [18] reported on the successful implementation of a healthy lifestyle programme in rural settings, noting the importance of organisational and local stakeholder involvement and buy-in to the programme. In addition, Damschroder et al. [19, 20] found that the dissemination of a weight management programme in Veterans Affairs Hospitals was strongly dependent on organisational characteristics, and that, in some situations, local champions could overcome organisational barriers. This is similar to the results from this study, which indicate that the social infrastructure is a critical factor for overcoming potential barriers in either the knowledge or physical infrastructures.

The results and recommendations presented were developed inductively from surveys conducted with Parks and Recreation leaders and key informant interviews of Parks and Recreation leaders and community members. The structure of the survey and interview guides were based on key domains from the CFIR, which allows us to situate our findings in the broader theoretical context of implementation research. The CFIR has been applied to implementation science in a variety of contexts (e.g. healthcare delivery and process re-design, quality improvement, health promotion and disease management) and health outcomes (e.g. mental health, obesity and blood pressure). The CFIR is most commonly applied to gain an in-depth understanding of practitioners’ experiences (e.g. implementation processes, barriers and facilitators to implementation) in innovation implementation [5]. Previous work has most frequently applied the CFIR to data collection and analysis post implementation [5]. Our use of the CFIR varied from this typical application in that we used the CFIR framework to evaluate compatibility in a pre-implementation assessment. In this context, we identified the knowledge, physical and social infrastructure as key domains for evaluation, which fit nicely into existing domains from the CFIR. Namely, the outer setting of the CFIR was most consistent with our ‘social infrastructure’, where agreement between community and organisational priorities was a key driver of successful implementation. Other domains from the CFIR were also particularly relevant to this implementation context, including intervention characteristics (i.e. whether the organisational leadership perceived this programme as evidence based), the inner setting (i.e. whether there were adequate knowledge and physical infrastructure to implement the programme), the characteristics of individuals (i.e. how closely community members identified with their local Parks and Recreation department), and the process (i.e. whether there were key opinion leaders in the Parks and Recreation department who could champion the programme). Utilisation of the CFIR in both the pre-implementation assessment and post-implementation measurement would be a next step in dissemination science.

This study had several limitations. Even though this study was conducted in four communities with significant sociodemographic diversity, the small number of communities that participated may mean that the findings are not generalisable to a wide range of community contexts. In particular, Parks and Recreation departments who engaged in this project already demonstrated significant commitment to health in their communities, and were willing to develop strategies to overcome barriers to implementation. For other communities where change is more difficult to achieve, these types of implementation strategies may not be as effective. However, we would posit that interest in adoption is the pre-requisite for effective implementation and dissemination efforts. The sample size at each of the Parks and Recreation centres was also relatively small, though it was reflective of the department. Consequently, it was not possible to determine if theme saturation was achieved during key informant interviews. Finally, all of the participants were selected based on their willingness to participate, which may have led to biased results, whereby individuals were pre-disposed to want this programme to succeed. Consequently, we may not have had access to a wider variability in organisational or community opinions.

## Conclusion

Using a comprehensive pre-implementation assessment of the knowledge, physical and social infrastructures in a community is an essential step in effective dissemination for community-based behavioural interventions. CFIR provides an instructive framework prior to implementation that allow for assessment of readiness and evaluation of alignment in community settings. Our research found that, when evaluating readiness and alignment, a responsive social infrastructure could provide the capacity to overcome potential barriers to implementation in either the knowledge or physical infrastructures.

## Additional files

Additional file 1: Interviews for Parks and Recreation programme staff and families. (DOCX 109 kb)

Additional file 2: Coding scheme for interviews. (DOCX 69 kb)

## Abbreviations

CFIR: Consolidated Framework of Implementation Research
