# Supplementary material for: Community readiness assessment for obesity research: pilot implementation of the Healthier Families programme
Source: Health Res Policy Syst. 2018 Jan 15;16:2. doi: 10.1186/s12961-017-0262-0 (PMC5769556; doi:10.1186/s12961-017-0262-0)
Supplement: Supplementary file 1 — Interviews for Parks and Recreation programme staff and families. (DOCX 109 kb) [file 12961_2017_262_MOESM1_ESM.docx]

*Additional File 1: Interviews for Parks and Recreation Program Staff and Families.*

# General Questions:

1. What type of programming do you offer for both parents and children together?
2. What type of programming do you offer to parents and preschool age children (ages 3- 5)?
3. If “yes” for either one or two, then ask:
   1. Tell us some of the successes that you have had in reaching out to families with preschool children?
   2. What are the barriers that you have confronted in engaging families with preschool children?

If “no”, then ask:

- 1. What haven’t you offered these programs in the past? (probe: interest, funding, organizational support, vision/mission)

1. How would family programming, designed for young parents and children together, fit with the goals of your parks and recreation center?

**Describe the Healthier Families Program then ask these questions:**

1. Family Outreach/Marketing: What outreach methods have you used to engage parents and young children in programing? Do you think any of these methods would work for the Healthier Families Program?
   1. If not, how would you let parents know about this program?
   2. What problems might you have in reaching families with young children?
   3. What do you think the barriers might be to getting parents to agree to take part in a weekly program?
2. Infrastructure Feasibility: How would you work a program like this into your physical facilities?
3. What would you use as a meeting space?
4. Would you be able to have child activities at the same time that the parents are learning? If not, why not?
5. Do you have space where you could offer cooking demonstrations?
6. Content: How do you think your staff would perceive delivering an exercise and healthy eating program for families with preschool children?
7. What would make them enthusiastic about delivering a program like this?
8. Are there aspects or elements of this they might perceive negatively?
9. How can we present this program to staff in your facilities so as to make them as enthusiastic as possible?
10. How would we train them to deliver this type of programming?
11. Which stakeholders in your community should we engage in this process? What is the best way to engage them?
12. What concerns or worries do you have around planning and implementing the ADAPT program in your community?

# Interview for Families

**General Questions:**

1. What activities in your community do you and your preschooler enjoy doing together?
   1. Where are these programs held?
   2. What makes them work? What benefit do you get from participating?
   3. What makes them most engaging?
2. How do you and your family use your local parks and recreation centers? If “yes”, ask:
   1. How do you involve your preschooler when you use parks and recreation centers?
   2. What types of family programming would you like to see at your Parks and Recreation center?

If “no”, ask:

- 1. Why not?

# Describe the Healthier Families Program then ask these questions:

1. Family Interest: How interested would you be in participating in this program on a scale of 1-5 (5 being “very interested” and 1 being “not at all interested”? (Probe to understand level of interest.)
   1. What would the challenges to participation be?
   2. What would you be most excited about?
2. Program Feedback: Show the 12 session topics and ask, “ How interested would you be about learning about (insert each topic here)?” and “How willing would you be to learn this from trained parks and recreation staff?”
3. Feasibility of Participation: How likely would you be to participate in a 12-week, weekly program for you and your preschooler?
   1. What would make it easy?
   2. What would make it hard? (Probes: transportation, time, other family responsibilities, work)
   3. What could parks and recreation provide to make participating easier for you?
4. What concerns or worries do you have around this type of program?
