# Supplementary material for: Community readiness assessment for obesity research: pilot implementation of the Healthier Families programme
Source: Health Res Policy Syst. 2018 Jan 15;16:2. doi: 10.1186/s12961-017-0262-0 (PMC5769556; doi:10.1186/s12961-017-0262-0)
Supplement: Supplementary file 2 — Coding scheme for interviews. (DOCX 69 kb) [file 12961_2017_262_MOESM2_ESM.docx]

*Additional File 2: Coding Scheme for Interviews*

Coding scheme developed inductively for assessment of key-informant interviews.

| **Code** |
| --- |
| 1. Characteristics of the community |
| 1. Characteristics of the Park and Recreation Center    1. Facilities    2. Staffing |
| 1. Specific programming    1. Adult programming    2. Child programming    3. Family programming |
| 1. Success stories |
| 1. Barriers    1. Money    2. Competing activities    3. Motivation    4. Stress    5. Transportation |
| 1. Perceptions of the Healthier Families program    1. Outreach/Marketing    2. Infrastructure    3. Staff perceptions    4. Working with staff    5. Training    6. Community outreach/engagement    7. Program Content |

| **Codes for family Interviews** |
| --- |
| 1. Perceptions of the Healthier Families program    1. Locations of activities    2. Perceived benefits |
| 1. Use of parks |
| 1. Family programming you would like to see |
| 1. Rating scales 2. How interested 3. Learning |
| 1. Willing to learn |
| 1. Likely to participate in 12-week program |
| 7) Barriers |
| 8) Healthier Families Program   - 1. Positive   2. Concerns |
| 9) Ideas |
